# Intraoperative Fraction of Inspired Oxygen and Lung Injury in Coronary Artery Bypass Grafting: Study Protocol for a Randomised Controlled Trial

**DOI:** 10.5152/TJAR.2023.22974

**Published:** 2023-04-01

**Authors:** Kaicheng Song, Sheng Wang, Jiange Han, Luyang Jiang, Junmei Xu, Ozan Akca, Kandis Adkins, Heidi M. Koenig, Mark S. Slaughter, Sean P. Clifford, Yuguang Huang, Jiapeng Huang

**Affiliations:** 1 Department of Anesthesiology and Perioperative Medicine University of Louisville, Louisville, KY, USA; 2 Department of Anesthesiology, Peking Union Medical College, Beijing, PR China; 3 Department of Anesthesiology, Guangdong Provincial People’s Hospital, Guangdong Academy of Medical Sciences, Guangzhou, China; 4 Department of Anesthesiology, Tianjin Chest Hospital, Tianjin, China; 5 Department of Anesthesiology, Peking University People’s Hospital, Beijing, China; 6 Department of Anesthesiology, The Second Xiangya Hospital, Central South University, Changsha, China; 7 Department of Anesthesiology and Critical Care Medicine, Johns Hopkins University, Baltimore, USA; 8 Department of Cardiovascular & Thoracic Surgery, University of Louisville, Louisville, KY, USA; 9 Department of Pharmacology and Toxicology, University of Louisville, Louisville, KY, USA; 10 The Center for Integrative Environmental Health Sciences, University of Louisville, Louisville, Kentucky, USA; 11 Division of Infectious Diseases, Center of Excellence for Research in Infectious Diseases (CERID), Department of Medicine, University of Louisville, Louisville, Kentucky, USA

**Keywords:** Cardiovascular and thoracic anaesthesia, coronary artery bypass grafting, lung injury, oxygen, perioperative care

## Abstract

**Objective:**

Postoperative pulmonary complications are a series of disorders that can contribute to respiratory distress and prolonged mechanical ventilation postoperatively. We hypothesise that a liberal oxygenation strategy during cardiac surgery leads to a higher incidence of postoperative pulmonary complications than a restrictive oxygenation strategy.

**Methods:**

This study is a prospective, observer-blinded, centrally randomised and controlled, international multicentre clinical trial.

**Results:**

After obtaining a written informed consent, 200 adult patients undergoing coronary artery bypass grafting will be enrolled and randomised to receive either restrictive or liberal oxygenation perioperatively. The liberal oxygenation group will receive 1.0 fraction of inspired oxygen throughout the intraoperative period, including during cardiopulmonary bypass. The restrictive oxygenation group will receive the lowest fraction of inspired oxygen required to maintain arterial partial pressure of oxygen between 100 and 150 mmHg during cardiopulmonary bypass and a pulse oximetry reading of 95% or greater intraoperatively, but no less than 0.3 and not higher than 0.80 (other than induction and when the oxygenation goals cannot be reached). When patients are transferred to the intensive care unit, all patients will receive an initial fraction of inspired oxygen of 0.5, and then fraction of inspired oxygen will be titrated to maintain a pulse oximetry reading of 95% or greater until extubation. The lowest postoperative arterial partial pressure of oxygen/fraction of inspired oxygen within 48 hours of intensive care unit admission will be the primary outcome. Postoperative pulmonary complications, length of mechanical ventilation, intensive care unit stay, hospital stay, and 7-day mortality after cardiac surgery will be analysed as secondary outcomes.

**Conclusion:**

This is one of the first randomised controlled observer-blinded trials that prospectively evaluates the influence of higher inspired oxygen fractions on early postoperative respiratory and oxygenation outcomes in cardiac surgery patients using cardiopulmonary bypass.

**Main Points**

- Postoperative pulmonary complications are a series of disorders that can contribute to respiratory distress and prolonged mechanical ventilation postoperatively.
- This is the study design of a prospective, observer-blinded, centrally randomised and controlled, international multicentre clinical trial to evaluate the influence of higher inspired oxygen fractions on early postoperative respiratory and oxygenation outcomes in cardiac surgery patients using cardiopulmonary bypass.
- Implementation of this clinical trial will help answer important questions on oxygen use in cardiac surgery.

## Introduction

Postoperative pulmonary complications (PPCs) include acute respiratory distress syndrome (ARDS), atelectasis, pulmonary oedema, aspiration, and pneumonia, which may lead to persistent lung injury, respiratory distress, and prolonged mechanical ventilation after surgery. Almost all patients undergoing cardiac surgery with cardiopulmonary bypass (CPB) develop some degree of postoperative pulmonary dysfunction, and 10%-25% patients develop PPCs.^1^ Postoperative pulmonary complications significantly worsen prognosis and result in prolonged recovery periods in the intensive care unit (ICU) and length of hospital stay.^2^ Although preoperative chronic obstructive pulmonary disease (COPD), smoking, anaemia, advanced age, and weakness impacted the incidence of PPCs, multiple intraoperative and postoperative factors such as mechanical ventilation, surgical manipulation, CPB, transfusion, lung recruitment manoeuvres, anaesthetic agents, and high-level fraction of inspired oxygen (FiO_2_) have been suggested as potential contributors to lung injury and PPCs.^3^

A postoperative protective or noninvasive mechanical ventilation strategy with low tidal volume [6 mL kg^–1^ ideal body weight (IBW)], lower driving pressures, and positive end-expiratory pressure (PEEP) is widely accepted as a key method for lung protection.^4^ However, intraoperative protective ventilation strategies are inconsistent and controversial during cardiac surgery, which suggests potential confounding factors may account for these results.^5^ Many critically ill cardiac surgical patients receive 1.0 FiO_2_ as a precaution to prevent hypoxia; thus, hyperoxia is common during cardiac surgery with a wide range of oxygen settings. Oxygen can directly and indirectly injure organs in 3 ways: (1) hyperoxia-related cardiovascular dysregulation, such as bradycardia and increasing vascular resistance; (2) direct tissue injury from increased production of reactive oxygen species (ROS); and (3) enhancement of ischaemia–reperfusion injury.^6^ Several clinical studies suggest that hyperoxia can cause myocardial injury, acute kidney injury (AKI), and neurologic dysfunction after cardiac surgery.^6^

Lungs can be adversely affected by hyperoxia as 1.0 FiO_2_ has been implicated in the development of atelectasis after even a short period. Prolonged ventilation with high concentrations of oxygen (FiO_2_ > 0.9) reliably causes severe hyperoxic acute lung injury and can be fatal when continued for extended periods. The severity of injury is proportional to both arterial partial pressure of oxygen (PaO_2_) (above 450 mmHg or FiO_2_ of 0.6) and duration of exposure.^7^ Hyperoxia during CPB was found to cause a greater decline in vital capacity and forced expiratory volume 5 days postoperatively when compared with normoxia.^8^ A retrospective analysis of 246 patients undergoing cardiac surgery with varying degrees of hyperoxia during mechanical ventilation (mild: PaO_2_ 100-199 mmHg, moderate: PaO_2_ 200-299 mmHg, severe: PaO_2_ > 300 mmHg) found that the group exposed to severe hyperoxia had more infectious complications, increased length of hospital stay, and increased time on mechanical ventilation after cardiac surgery.^9^ Another retrospective study consisting of 73 992 patients revealed that the median intraoperative FiO_2_ was associated with increased risk of respiratory complications and 30-day mortality.^10^ On the contrary, a retrospective study of 5056 patients found that intraoperative hyperoxia did not change the postoperative pulse oximetry reading (SpO_2_)/FiO_2_ ratio or the risk for pulmonary complications.^11^ Due to the contradictory nature of these retrospective studies, carefully designed prospective studies are needed to explore the relationship between FiO_2_ and PPCs after cardiac surgery. We hypothesise that a liberal oxygenation strategy during coronary artery bypass grafting (CABG) surgery leads to higher incidence of pulmonary complications than restrictive oxygenation strategy. The primary outcome is the lowest postoperative PaO_2_/FiO_2_ within the designated blood gas analysis testing periods in the first 48 hours of ICU admission comparing liberal (FiO_2_ 1.0) and restrictive intraoperative oxygenation (lowest FiO_2_ with adequate oxygenation) groups. Postoperative pulmonary complications, length of mechanical ventilation, ICU stay, hospital stay, and 7-day mortality after cardiac surgery will be analysed between liberal and restrictive oxygenation groups as secondary outcomes.

## Methods

### Study Design and Setting

**Figure 1.**
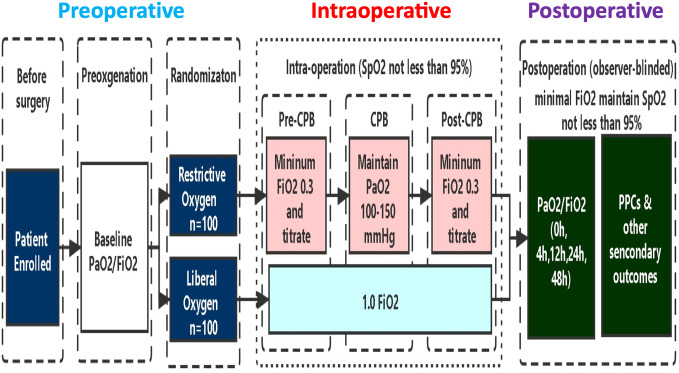
Algorithm and timetable for enrolment, allocation to restrictive or liberal oxygenation groups, intraoperative management, and postoperative data collection.

This study is a prospective, observer-blinded, web-based centrally randomised, controlled, multicentre, international clinical trial to assess the relationship between intraoperative FiO_2_ and PPCs. Surgical technique will be restricted to CABG with CPB through median sternotomy to minimise variation. Arterial partial pressure of oxygen/FiO_2_ will be assessed at baseline (preoperative values), time of postoperative ICU admission as well as 4 hours, 8 hours, 12 hours, 18 hours, 24 hours, 36 hours, and 48 hours after postoperative ICU admission (Figure 1). The study will be conducted at University of Louisville Health, Louisville, KY, USA, and 5 teaching hospitals from China with a combined more than 8000 open-heart surgeries and over 1000 CABG with CPB cases per year. We have been assured fair access to research subjects to enable completion of this study within a 1-year period. Written informed consent will be obtained from each participant preoperatively.

This study is designed not to interfere with routine clinical workflow, and patients will be centrally randomised to either restrictive or liberal oxygenation groups in a 1 : 1 ratio, and the randomisation will be stratified for each centre. For blinding, the research fellows and ICU physicians who record and assess the primary and secondary outcomes as well as who manages the patients will be blinded to the randomisation group assignment. The principal investigator (PI) of each centre and the physicians who conduct the anaesthesia and monitoring intraoperatively in the operating room are unblinded due to necessary requirements for clinical care. This protocol is in accordance with the Recommendations for Interventional Trials (SPIRIT) checklist. The SPIRIT figure demonstrates the schedule of enrolment, interventions, and outcome assessment of this trial (Table 1).

### Study Registration

The Institutional Review Board (IRB) at the University of Louisville approved the study protocol for University of Louisville sites and provides ongoing oversight (IRB 19.0988). All sites from China will obtain approvals from local IRBs. Data sharing agreements will be obtained from these study sites. This study was registered at ClinicalTrials.gov on 3 October 2019 (NCT04115501). The trial has not begun recruitment pending IRB approval from Chinese sites. All amendments to this protocol will be reported to the IRB of University of Louisville as well as the relevant IRBs of involved study sites.

### Inclusion and Exclusion Criteria

The study will enrol adult patients aged 18-80 with a body mass index (BMI) from 20 to 39.9 who are undergoing isolated CABG with CPB support through median sternotomy with subsequent admission to an ICU. Patients with severe COPD (i.e., Forced expiratory volume in 1 second (FEV_1_) < 50% and preoperative PaCO_2_ > 60 mmHg) will be excluded. Pregnant women will be excluded. Patients with recent acute coronary syndromes (<1 week), acute onset of heart failure, severe anaemia (haemoglobin < 8g dL^–1^), preoperative supplemental oxygen to maintain oxygen saturations (SpO_2_) above 90%, right to left intracardiac shunts, unresolved carotid stenosis defined as >50% stenosis of either carotid artery, end-stage renal disease requiring dialysis, recent smoking (within 1 week), and planned regional anaesthesia will be excluded (Table 2).

### Intervention and Anaesthetic Regimen

Anaesthesia will be induced with a combination of sufentanil (1-4 μg kg^–1^) or fentanyl (2-10 μg kg^–1^), propofol (1-3 mg kg^–1^) or etomidate (0.2-0.3mg kg^–1^), and succinylcholine (1-2mg kg^–1^) or rocuronium (1-2 mg kg^–1^). General anaesthesia will be maintained with sevoflurane or isoflurane (Minimum alveolar concentration [MAC] 0.5-1.5). Additional narcotics and muscle relaxants will be used at the discretion of attending anaesthesiologists before and after CPB. The total dose of sufentanil is not to exceed 15 μg kg^–1^ and the total dose of fentanyl is not to exceed 10 μg kg^–1^, and only 1 narcotic will be used for any single case. Train of 4 will be monitored during the entire case to adjust the dosage of muscle relaxants. Arterial blood gas (ABG) will be obtained in the pre-anaesthesia clinic while breathing room air, after anaesthesia induction but before CPB, on CPB, after CPB, and other clinically indicated time points during cardiac surgery to adjust FiO_2_ in the restrictive oxygenation group. While the patient is on CPB, anaesthesia circuits will be disconnected and lungs will be exposed to the atmosphere. Before coming off CPB, recruitment manoeuvre will be performed to inflate lungs. When the patient is transferred to the ICU, ABG analysis will be performed on admission and at 4 hours, 8 hours, 12 hours, 18 hours, 24 hours, and 48 hours after ICU admission. Tidal volume will be set at 6-8 mL kg^–1^ IBW, respiratory rate to maintain normocapnia, PEEP will be managed between 5 and 10 cm H_2_O at the discretion of the intensivist, and I : E ratio between 1:1.5 and 1:2.

Postoperative pain management will be provided as a bolus of 25 μg fentanyl, 0.2 mg hydromorphone intravenously, a continuous opioid infusion as needed, or 5-10 mg hydrocodone or oxycodone with acetaminophen orally with the aim of a visual analogue score less than 4. The goal is to achieve adequate pain control without respiratory compromise. Only normal saline, lactated Ringer’s solution, human albumin, or cell saver blood are allowed as maintenance infusion fluids during and after surgery. Fluid infusion rate from CPB weaning until the end of surgery should be less than 10 mL kg^–1^ h^–1^ to avoid pulmonary oedema; otherwise, the patient will be withdrawn from analysis. The trigger for transfusion of allogeneic red blood cells is 7 g dL^–1^ during CPB and 8 g dL^–1^ while not on CPB. The trigger for transfusion of plasma, cryoprecipitate, and platelets is determined by the laboratory values and clinical protocols. Transfusion of more than 2 units of red blood cells (RBCs), fresh-frozen plasma, platelets, or cryoprecipitate within 48 hours of ICU admission will result in withdrawal from the study.

After anaesthesia induction and intubation, the restrictive oxygenation group will have FiO_2_ set at a minimum of 0.3 to maintain SpO_2_ greater than or equal to 95%. If the SpO_2_ is above 95%, a 10% further reduction in FiO_2_ will be attempted to achieve the lowest possible FiO_2_ while maintaining an SpO_2_ > 95%. During CPB, a blended air/oxygen mixture will be titrated to ABG results with the goal of PaO_2_ between 100 and 150 mmHg. The liberal oxygenation group receives 1.0 FiO_2_ throughout the intraoperative period, including during CPB. After admission to ICU, all patients will receive 0.5 FiO_2_ initially and then titrated to the lowest FiO_2_ while maintaining SpO_2_ ≥ 95% until extubation. Criteria for sedation weaning include a stable heart rate and blood pressure and a chest tube output < 200 mL over the last 2 hours. Criteria for mechanical ventilation weaning trial include a rapid shallow breathing index <100, a respiratory rate < 35, an SpO_2_ > 92%, a heart rate < 140 bpm, a systolic blood pressure <180 mmHg and >90mmHg, and no anxiety or diaphoresis from increased work of breathing. Goal-directed extubation criteria will be utilised in the cardiac ICUs of study centres. These criteria include an FiO_2_ ≤ 0.40, an SpO_2_ ≥ 95%, a rapid shallow breathing index < 100, and a negative inspiratory force ≥ –20cm H_2_O. Postoperative narcotic analgesia and sedation approaches are standardised to minimise the discrepancies among the centres. After extubation, the patient may still need continuous oxygen support in the ICU or after ICU discharge, and the lowest SpO_2_/FiO_2_ ratio will be recorded daily until discharge from the hospital.

### Safety Assessment

The PI, investigators, study coordinators, attending anaesthesiologists, and intensivists will be in close communication throughout the entire procedure until patient discharge to ensure protocol adherence. Patients will be withdrawn intraoperatively if they require 1 lung ventilation, additional procedures other than CABG, circulatory arrest, repeated episodes of CPB support, or usage of circulatory assist devices. If a patient suffers an SpO_2_ < 90% for more than 5 minutes at any time during the surgery, the patient will be withdrawn, and this will be recorded as a serious adverse event. Postoperatively, if a patient experiences delayed extubation (i.e., weaning/extubation period lasting longer than 48 hours) or requires reintubation for non-respiratory reasons (i.e., surgical reasons), suffers major bleeding (i.e., requiring a delay in extubation due to haemodynamic management), and develops heart failure, they will be withdrawn. If there is a perioperative allogenic RBC transfusion of more than 2 units or if a patient receives any other blood products, the patient will also be withdrawn (Table 2). Other postoperative events such as AKI, stroke, myocardial infarction, reoperation, and sternal wound infection will be recorded as adverse events and be analysed to determine whether they are related to the intervention. A trained research team member (i.e., fellow, study coordinator) will monitor the protocol compliance and report any adverse events to the IRB.

### Interim Analysis and Stopping Rule

A data and safety monitoring board (DSMB) consisting of members who are not involved in the study and have expertise in clinical aspects of the illnesses, surgery, study patient population, clinical trial conduction, methodology, and biostatistics will analyse the data of all enrolled subjects after enrolment of each of the 40 study patients. A DSMB will have the full authority to determine the need to terminate this study early. The stopping rule will take effect if the mean value of minimal PaO_2_/FiO_2_ (primary outcome) in restrictive oxygenation group is clinically concerning lower (PaO_2_/FiO_2_ < 150 mmHg for 2 consecutive measurement periods). There will be 4 review periods for each of the 40 new patients after the completion of the data collection period.

### Statistical Analysis

#### Sample Size Calculation

A decrease of 10% in PaO_2_/FiO_2_ from the baseline is considered as clinically significant. The ARDS definition is PaO_2_/FiO_2_ less than 300 mmHg; thus, a difference of 30 mmHg of mean value of the lowest PaO_2_/FiO_2_ postoperatively between the 2 groups will be considered as clinically significant. Assuming SD of 60 mmHg, *α* = 0.05, and 80% power with 2-sided *t*-test, at least 160 subjects will be needed in total to detect a difference (80 patients in each group). Considering potential withdrawal, protocol violation, and failure to follow-up, we will aim to recruit 200 patients (100 patients in each group) for this study.

### Data Analysis

Data will be expressed as mean ± SD or median (minimum and maximum) and percentage as appropriate for continuous and categorical variables, respectively. A *t-*test will be used to compare continuous variables. For comparison of ratio or ordinal categorical data, the Pearson chi-squared test (or Fisher’s exact) or a Mann–Whitney *U*-test will be used. Two-sided *P-*values less than .05 will be considered statistically significant. All the analyses will be conducted using the R software.

The primary outcome of the study is the lowest PaO_2_/FiO_2_ within 48 hours after ICU admission between liberal and restrictive oxygenation groups. A non-parametric equivalent *t-*test will be used to compare groups. The percentage of shift from baseline will also be calculated and compared by the *t-*test. The ratio of shift over 10% from the baseline of each group will be compared by the chi-squared test. Secondary outcome analysis of PPC incidence and mortality of the groups will be compared by the chi-squared test. Odds ratios or measures of relative risk will include 95% CIs. Secondary outcomes including the length of mechanical ventilation and lengths of postoperative ICU and hospital stay will be calculated and compared by a *t-*test.

### Expected Results

#### Primary Endpoint

The PaO_2_/FiO_2_ is classified into 3 categories of severity: severe (≤100 mm Hg), moderate (>100-≤200), and mild (>200-300). The PaO_2_/FiO_2_ is frequently calculated and considered a reflection of lung injury, and low PaO_2_/FiO_2_ is associated with more PPCs and mortality. Previous studies revealed that a PaO_2_/FiO_2_ decrease of 10% from baseline was clinically meaningful. The investigators note the lowest PaO_2_/FiO_2_ within 48 hours after ICU admission as the primary outcome and take a difference of 30 mmHg (considering the normal value >300 mmHg) between the 2 groups as clinically significant.

### Secondary Endpoints

The European taskforce published guidelines for perioperative clinical outcomes (EPCO) (Table 3)^12^; PCCs after surgery include ARDS, noncardiogenic pulmonary oedema, pulmonary infection, pneumonia, pleural effusion, atelectasis, and respiratory failure. The composite of these events is considered as the secondary endpoint of this study. Other secondary outcomes will include the duration of mechanical ventilation, ICU and hospital stay, and patient mortality within 7 postoperative days. Time-weighted average of PaO_2_/FiO_2_ ratio within the first 48 hours will also be followed as an additional outcome.

### Data Collection

Clinical and demographic variables, including age, BMI, gender, ejection fraction, comorbidities, and echocardiographic assessments, will be collected. Arterial blood gas will be collected in the preanaesthesia clinic, before incision and CPB, on CPB, and after CPB until skin closure. Arterial blood gas will also be performed at ICU admission (as 0 hour postoperatively) and at 4 hours, 8 hours, 12 hours, 18 hours, 24 hours, 36 hours, and 48 hours after ICU admission. Ventilatory parameters that will be recorded include FiO_2_, tidal volume, respiratory rate, PEEP, I : E ratio, pH, PaO_2_, PaCO_2_, HCO_3_, BE, and SpO_2_. Every patient will get a chest x-ray upon postoperative ICU admission as baseline. All pulmonary complications will be diagnosed based on the EPCO definitions. Intensivists who will assess, collect, and analyse data are blinded to the group assignment.

All collected information will be recorded in an electronic case report form (eCRF). After de-identification of all data, eCRFs will be securely stored at an online database. Trained research fellows will be responsible for building and maintaining the eCRFs. Each PI is responsible for monitoring the data entry for completeness, timeliness, and accuracy. For PPC diagnosis, training to all assessors will be provided, and interrater reliability checks between the assessors will be performed.

### Reporting of Compliance and Adverse Events

A trained research fellow/study coordinator will monitor protocol compliance, occurrence of protocol violation, and reporting of adverse events to the IRB.

### Follow-up

Subjects will be contacted via telephone by trained study team members on postoperative day 7 if the patient is already discharged from the hospital at that time.

## Discussion

There are 500 000 cardiac surgeries a year in the USA alone. Almost every patient has lung injury, and up to 25% of them develop PPCs after cardiac surgery.^13^ Most of prior studies focus on postoperative mechanical ventilation strategies and incidence of PPCs and mortality in the ICU.^14,15^ Postoperative pulmonary complications are mainly attributed to ventilation-induced lung injury (VILI) and ventilator-associated pneumonia (VAP). Both VILI and VAP are highly time dependent and are most common in patients who are ventilated for longer than 48 hours postoperatively. A protective mechanical ventilation strategy or an adaptive support ventilation triggered by spontaneous breathing is wildly accepted and applied to wean patients in the ICU.^15, 16^ Nowadays, patients are usually extubated within 6 hours postoperatively after an uneventful CABG. Therefore, the intraoperative mechanical ventilation has become an important proportion of overall mechanical ventilation.^17,18^ Therefore, the intraoperative ventilatory management might have significant impacts on outcomes after CABG even more than the ICU management.

Several studies suggest that a protective ventilation strategy is beneficial and can reduce PPCs, but some studies found no improvement or even increased mortality in cardiac patients with the protective ventilation strategies.^5,19^ The most significant ventilation parameter change between intra- and postoperative period is FiO_2_, and scarce data currently exist to evaluate the relationship between intraoperative FiO_2_ and PPCs in cardiac surgery.

Previous studies on FiO_2_ have diverse definitions of hyperoxia. Some studies’ criteria for hyperoxia are in the normoxia range for other studies.^20^ There are no well-established PaO_2_ targets on CPB, and clinical practice is highly variable. Based on the oxygen haemoglobin dissociation curve of normal subjects, SpO_2_ decrease from 100% to 90% will decrease PaO_2_ by 40 mmHg. We choose a minimum SpO_2_ of 95% as our safe threshold because it is close to PaO_2_ 80mmHg. The PaO_2_ from ABG is more accurate and reliable but cannot be monitored continuously; thus SpO_2_ is a more feasible and appropriate index for FiO_2_ adjustments.

The major limitation of this study lies in the difficulty of balancing many confounding factors during CABG with CPB that are harmful to the lungs, including the surgical procedure, CPB, hypothermia, blood contact with artificial surfaces, ischaemia–reperfusion injury, administration of blood products, and lung collapse.^21^ In our protocol, if the patient develops haemodynamic instability which requires a return to CPB or suffers circulatory arrest, they will be withdrawn from the study. We will also withdraw patients who require mechanical circulatory support systems like extracorporeal membrane oxygenation, ventricular assist device, or an Impella device for similar reasons. Transfusion is common in cardiac surgery, but CABG has a relatively low transfusion rate compared to other open-heart surgeries.^22^ Transfusion-associated lung injury (TRALI) has been well defined, and it is a prominent factor for increased morbidity and mortality.^23,24^ The reported incidence of TRALI varies widely, ranging from 0.0008% to 1.2% per unit of blood product administered and from 0.08% to 8% per transfused patient.^25^ Since transfusion of blood products, in particular blood plasma, may cause lung injury, these patients will also be withdrawn from our analysis. We predict a high rate of withdrawal, not only for safety reasons but also for bias control and balancing of confounding factors. Another study limitation is our inability to blind the anaesthesiologist. Only ICU physicians and research fellows/study coordinators who assess the outcomes will be blinded.

There exists increasing evidence that a restrictive oxygenation strategy may be beneficial in various groups of patients.^26^ It is important to understand the relationship between FiO_2_ and lung injury after cardiac surgery. This is one of the first randomised controlled trials to prospectively evaluate the influence of high inspired oxygen concentrations on pulmonary outcomes and oxygenation after cardiac surgery.

## Figures and Tables

**Table 1.** The Schedule of Enrolment, Interventions, and Outcome Assessment in Different Periods of This Trial

	Enrolment	Allocation	Post-allocation			Close-out
Timepoint	Preoperative 1 Day Before Surgery	Before Oxygenation	Intraoperative	Postoperative 0 hours, 4 hours, 8 hours,12 hours, 24 hours, 48 hours	Discharge	Postoperative 7 Days
Enrolment						
Eligibility screen	**X**					
Informed consent	**X**					
Allocation		**X**				
Interventions						
Restrictive oxygenation group			**X**			
Liberal oxygenation group			**X**			
Assessments						
PaO_2_/FiO_2_ (ABG)		**X**	**X**	**X**		
Postoperative pulmonary complications				**X**	**X**	
Length of mechanical ventilation					**X**	
ICU stay					**X**	
Hospital stay					**X**	
Mortality						**X**

ABG, arterial blood gas; ICU, intensive care unit.

**Table 2.** Inclusion, Exclusion, and Withdrawal Criteria

Inclusion and Exclusion Criteria	Withdrawal Criteria
Inclusion criteria	Preoperative
Age from 18 to 80 BMI from 20 to 39.9 Isolated CABG CPB planned Median sternotomy approach Subsequent admission to ICU	Onset of heart failure Onset of acute decompensated arrhythmia Onset of low oxygen saturation (SpO_2_ < 90%) on supplemental oxygen
Exclusion criteria Severe COPD (PaCO_2_ > 60 mmHg) Recent acute coronary syndrome (<1 week) Acute onset of heart failure Severe anaemia (haemoglobin < 8 g dL^–1^) Preoperative supplemental oxygen required to maintain SpO_2_ >90% Right to left intracardiac shunts Carotid stenosis defined as >50% stenosis of either carotid artery End-stage renal disease requiring haemodialysis Recent smoking (within 1 week) Planned regional anaesthesia	Intraoperative One lung ventilation Additional procedures other than CABG CPB more than once or circulatory arrest Fluid infusion rate more than 10 mL kg^–1^ h^–1^ post CPB Patients in restrictive oxygenation group needing FiO_2_ > 0.7 to maintain SpO_2_ ≥95% SpO_2_ < 90% lasting for more than 5 minutes
	Postoperative Non-respiratory reasons to reintubate Delayed extubation due to bleeding Delayed extubation due to heart failure	Any time Allogenic transfusion except less than 2 units RBC Circulatory assist device implantation (ECMO, VAD, or Impella^®^ device)

BMI, body mass index; CABG, coronary artery bypass graft; COPD, chronic obstructive pulmonary disease; CPB, cardiopulmonary bypass; ECMO, extracorporeal membrane oxygenation; ICU, intensive care unit; RBC, red blood cell; VAD, ventricular assist device.

**Table 3.** Definition of Postoperative Pulmonary Complications

Complications	Definitions or Diagnostic Method
Respiratory infection	Antibiotics for suspected infection with 1 or more of the following: new or changed sputum, new or changed lung opacities, fever, white blood cell count >12 × 109 L^–1^
Atelectasis	Lung opacification with mediastinal shift, hilum or hemidiaphragm shift towards the affected area, with compensatory hyperinflation in adjacent non-atelectatic lung
Pneumonia	Chest x-ray with at least 1 of the following: infiltrate, consolidation, and cavitation, plus at least 1 of the following: fever >38˚C with no other cause, white cell counts 12 × 10^9^ L^–1^, and >70 years of age with altered mental status with no other cause plus at least 2 of the following: new purulent/ changed sputum, increased secretions/suctioning, new/worse cough/dyspnoea/tachypnoea, rales/bronchial breath sounds, and worsening gas exchange
Noncardiogenic pulmonary oedema	Evidence of fluid accumulation in the alveoli with enough cardiac output
Pleural effusion	Chest x-ray with blunting of costophrenic angle, loss of sharp silhouette of the ipsilateral hemidiaphragm in upright position, displacement of adjacent anatomical structures, or (in supine position) hazy opacity in 1 hemithorax with preserved vascular shadows
Respiratory failure	Postoperative PaO_2 _< 60 mm Hg on room air
Acute respiratory distress syndrome	PaO_2_/FiO_2_ ratio < 300 mm Hg

Adapted from Jammer (2015).^12^
